# Inflammatory biomarkers and risk of breast cancer among young women in Latin America: a case-control study

**DOI:** 10.1186/s12885-022-09975-6

**Published:** 2022-08-11

**Authors:** Emma Fontvieille, Mathilde His, Carine Biessy, Anne-Sophie Navionis, Gabriela Torres-Mejía, Angélica Ángeles-Llerenas, Isabel Alvarado-Cabrero, Gloria Inés Sánchez, Edgar Navarro, Yorlany Rodas Cortes, Carolina Porras, Ana Cecilia Rodriguez, Maria Luisa Garmendia, José Luis Soto, Leonor Moyano, Peggy L. Porter, Ming Gang Lin, Jamie Guenthoer, Isabelle Romieu, Sabina Rinaldi, PRECAMA team, Jenny Tejeda, María Felix Lazcano, Libia Zulema Franco, Roberto Jaramillo, Alberto Angel, Carlos Andres Ossa, William H. Arias, Gabriel Bedoya, Alicia Cock-Rada, Carolina Echeverri, Fernando Herazo, Israel Díaz-Yunez, Angel Hernández, Bernal Cortes, Paula Gonzalez, Rebecca Ocampo, Diego Guillen, Viviana Loría, Catalina Vial, Lizette Diaz, Elizabeth Donato, Thomas Donn, Kelly Wirtala, Hailey Loucks

**Affiliations:** 1 International Agency for Research on Cancer (IARC/WHO), Nutrition and Metabolism Branch, Lyon, France; 2 Centre for Population Health Research, National Institute of Public Health, Cuernavaca, Mexico; 3 Servicio de Patología, Hospital de Oncología, CMN SXXI, Instituto Mexicano del Seguro Social, Ciudad de México, México; 4 Group Infection and Cancer, School of Medicine, University of Antioquia, Medellín, Colombia; 5 Grupo Proyecto UNI-Barranquilla, Universidad del Norte, Barranquilla, Colombia; 6 Hemato Oncologos, Cali, Colombia; 7 Agencia Costarricense de Investigaciones Biomédicas (ACIB)-Fundación INCIENSA, San Jose, Costa Rica; 8 Instituto de Nutrición y de Tecnología de los Alimentos, Universidad de Chile, Santiago, Chile; 9 National Institute of Cancer, Santiago, Chile; 10 Division of Human Biology, Fred Hutchinson Cancer Research Center, Seattle, USA; 11 Hubert Department of Global Health, Emory University, Atlanta, Georgia USA

**Keywords:** Inflammation, Biomarkers, Breast cancer, Premenopausal, Latin America

## Abstract

**Background:**

Breast cancer incidence is increasing rapidly in Latin America, with a higher proportion of cases among young women than in developed countries. Studies have linked inflammation to breast cancer development, but data is limited in premenopausal women, especially in Latin America.

**Methods:**

We investigated the associations between serum biomarkers of chronic inflammation (interleukin (IL)-6, IL-8, IL-10, tumor necrosis factor-α (TNF-α), interferon-γ (IFN-γ), leptin, adiponectin) and risk of premenopausal breast cancer among 453 cases and 453 matched, population-based controls from Chile, Colombia, Costa Rica, and Mexico. Odds ratios (OR) were estimated using conditional logistic regression models. Analyses were stratified by size and hormonal receptor status of the tumors.

**Results:**

IL-6 (OR_per standard deviation (SD)_ = 1.33 (1.11–1.60)) and TNF-α (OR_per SD_ = 1.32 (1.11–1.58)) were positively associated with breast cancer risk in fully adjusted models. Evidence of heterogeneity by estrogen receptor (ER) status was observed for IL-8 (P-homogeneity = 0.05), with a positive association in ER-negative tumors only. IL-8 (P-homogeneity = 0.06) and TNF-α (P-homogeneity = 0.003) were positively associated with risk in the largest tumors, while for leptin (P-homogeneity = 0.003) a positive association was observed for the smallest tumors only.

**Conclusions:**

The results of this study support the implication of chronic inflammation in breast cancer risk in young women in Latin America. Largest studies of prospective design are needed to confirm these findings in premenopausal women.

**Supplementary Information:**

The online version contains supplementary material available at 10.1186/s12885-022-09975-6.

## Introduction

Breast cancer is the most frequently diagnosed cancer worldwide and the leading cause of cancer death among women [1]. However, the age distribution of the cases varies geographically, with 12% of estimated breast cancer cases occurring in women younger than 45 years old in high-income countries, in contrast to 20% in Latin America [1]. This higher percentage of cases in young women in Latin America can only partially be explained by the different age structure of the population, and rather suggests that some risk factors, or the distribution of breast cancer subtypes, are characteristic of this population [2]. However, very few studies have been conducted so far to investigate the etiology of premenopausal breast cancer specifically in Latin America [3].

Obesity is associated with a decreased risk of breast cancer in premenopausal women in different populations worldwide [4], including in Latin American women [5, 6]. This inverse association is not well understood but may be associated to different metabolic phenotypes imperfectly captured by generic anthropometric measures, with varying degrees of excess body fat, dyslipidemia, insulin resistance, elevated blood pressure, and systemic low-grade inflammation [7, 8].

Systemic inflammation is a known correlate of obesity, as macrophages become the largest cell population in obese adipose tissue, resulting in altered expression of pro- or anti-inflammatory cytokines in the circulation [9]. In addition, increased fat mass is associated with altered production of adipokines [10], of which leptin and adiponectin are the most studied. These two peptides, produced by the adipose tissue, have various biological functions, including the regulation of satiety [10], and may be involved in insulin resistance [9].

Tight links exist between inflammation and the development of different cancers, including breast cancer [11]. A meta-analysis of 12 prospective studies conducted mostly in Caucasian women and focusing on C-Reactive protein (CRP), a non-specific marker of acute inflammation, showed a positive association with breast cancer risk [12]. However, only two studies were conducted in premenopausal women, with inconsistent findings [13, 14]. Studies considering biomarkers of chronic inflammation remain limited, in particular in young women [15]. To our knowledge, only one prospective study reported associations between inflammatory markers such as cytokines and breast cancer risk in premenopausal women [16], showing contrasting results in associations, while most of the studies published so far on this topic were mainly of clinical settings and relatively small sample sizes.

In this work, we examined associations between seven serum biomarkers of inflammation (interleukin 6 (IL-6), interleukin 8 (IL-8), interleukin 10 (IL-10), tumor necrosis factor α (TNF-α), interferon γ (IFN-γ) and adipokines (leptin and adiponectin) and breast cancer risk in premenopausal women from Latin America, using data from the multicentric PRECAMA population-based case–control study.

## Methods

### PRECAMA study

PRECAMA is an ongoing multicentric population-based case–control study based in Chile, Colombia, Costa Rica, Mexico, and Brazil [17–19]. Information on lifestyle, health and reproductive history was collected at recruitment. Standardized protocols were used to collect biological samples (fasting blood, spot urine) and anthropometric measures (height, weight, waist and hip circumferences) at the time of interview. Immunohistochemistry analyses on tumor tissue for estrogen receptor (ER), progesterone receptor (PR), human epidermal growth factor receptor 2 (HER2), epidermal growth factor receptor (EGFR) and cytokeratin 5/6 (CK5/6) was performed centrally at the Fred Hutchinson Cancer Research Center in Seattle. Tumour samples with ≥ 1% immunostained tumour cell nuclei were considered positive (ER positive (ER +), PR positive (PR +)). For HER2, samples were considered positive if there was strong membrane immunostaining (3 +).

All participants gave written informed consent before enrolment, and the study protocols were approved by the local institutional review and the International Agency for Research on Cancer (IARC).

### Selection of cases and controls

Cases were women diagnosed with first primary incident breast cancer in general or cancer-specific hospitals or private oncology institutes [19], recruited before receiving any treatment, who fulfilled the following inclusion criteria: 1) age 20–45 years; 2) being resident for ≥ 3 years in the same city district; 3) having an incident primary invasive breast cancer with positive biopsy and clinical staging; 4) having menstruated at least once in the past 12 months.

Controls were selected from the general population residing in the same city district as the case for at least 3 years using a multilevel sampling frame [19]. Controls were matched to cases on age (± 3 years), city district of residence, and health insurance institution.

Participants from Brazil were not included in the present work as the recruitment started later.

A total of 453 cases and 453 controls were included in the present analysis.

### Biological specimens’ collection and analysis

Biological specimens were collected according to standardized protocols [19–21]. Blood samples were obtained at recruitment, prior to any treatment, by venipuncture using vacutainers aliquoted into serum, plasma, red blood cells, and buffy coat and stored at -80 °C. Half of the aliquots were stored locally in each center while a mirror half was centralized and stored at IARC.

Laboratory measurements of IL-6, IL-8, IL-10, TNF-α, IFN-γ, leptin, and adiponectin were performed on serum samples at IARC (Biomarkers Group) using a highly sensitive and specific multiplexing electro-chemiluminescent assay (V‐PLEX™ Custom Human Cytokine Kit, Meso Scale Discovery, Rockville, MD). The laboratory technician who performed assays was blind to case–control status of the samples. Samples from cases and matched controls were analyzed in the same analytical batch. Three quality control samples were included in duplicate in each batch of analyses. Overall within-batch coefficients of variations ranged from 2.9% for IFN-γ to 9.7% for IL-6. Between-batch CVs ranged from 5.7% for adiponectin to 11.4% for IFN-γ. For each biomarker, values below the lower limit of quantification were imputed to this lower limit (*n* = 6 for IL-6; *n* = 44 for IL-10; *n *= 7 for TNF-α; *n* = 40 for IFN-γ) while values above the upper limit of quantification were imputed to this upper limit (*n* = 6 for leptin).

All cases and controls included in the study had available measurements for IL-6, IL-10, TNF-α, IFN-γ, and adiponectin, while leptin was available only for 860 subjects (430 case–control pairs), and IL-8 on 628 subjects (314 case–control pairs).

### Statistical analysis

Population’s characteristics were described using frequency for categorical variables, and arithmetic mean and standard deviation for continuous variables, except for biomarker concentrations which were described using geometric means.

In all following analyses, concentrations of biomarkers were log-transformed to approximate normal distribution and we used residuals of log-transformed variables regressed on analytical batch as exposures of interest. For covariates, missing values represented less than 5% of values and were imputed to the mode (parity) or to the median (waist circumference, hip circumference, height, weight, BMI, use of hormones at blood collection, and, among parous women only, age at first full-term pregnancy). Triple-negative (TN) tumors were defined as ER-negative, PR-negative, and HER2-negative. Among the TN tumors, basal-like tumors were defined as ER-negative, PR-negative, HER2-negative, and EGFR-positive (EGFR +) and/or CK5/6-positive (CK5/6 +).

Spearman's partial correlation coefficients between concentrations of inflammation markers (as residuals of log-transformed variables regressed on analytical batch) and anthropometric factors and age were calculated among controls, adjusting for age (except for correlations with age).

Odds ratios (ORs) for risk of breast cancer and their associated 95% confidence intervals (CI) were estimated using conditional logistic regression models. Concentrations of biomarkers and leptin/adiponectin ratio (residuals of log-transformed variables on analytical batch) were examined as continuous variables (OR estimated per standard deviation increment) and in quartiles, defined on the distributions of control participants. Tests for linear trends across quartiles were computed by assigning all individuals in a quartile the median value of the biomarker in this quartile and considering this variable as a continuous variable in the model. Potential non-linear associations were modelled using restricted cubic splines (3 knot), and BMI-adjusted models were compared by likelihood ratio test.

We considered four models for each biomarker. First, we evaluated associations in a model including only matching variables. Second, we evaluated a model adjusted for BMI (continuous). Third, we examined a model adjusted for waist circumference (continuous) instead of BMI, as these variables are highly correlated but reflect different types of adiposity. Eventually, to rule out confounding originating from other variables than BMI and waist circumference, we examined a model adjusted for the following breast cancer risk factors: BMI (continuous), age at menarche (years, continuous), number of full-term pregnancies (0/1/2/ ≥ 3), age at first pregnancy (nulliparous, tertiles), breastfeeding duration (nulliparous, tertiles), use of hormones at blood collection (yes/no), personal history of benign breast disease (yes/no), family history of breast cancer in first-degree relatives (yes/no), smoking status (ever smoker/never smoker), alcohol consumption (g/day), moderate physical activity (hours/day), education level (up to primary school/secondary school/longer than secondary school), and adult height (continuous).

In cases for whom the information was available, analyses were stratified according to immunohistochemistry results for ER, PR, and HER2 (298 pairs, ER-positive, ER-negative, triple-negative) and tumor size (N = 311 pairs, ≤ 2 cm/2–5 cm/ > 5 cm). All models in stratification were adjusted for BMI only, since sample size was too limited in subgroups to run the fully adjusted model. We additionally stratified analysis by BMI (< 25/ ≥ 25 kg/m^2^; < 30/ ≥ 30 kg/m^2^), using logistic regression analyses adjusted for matching factors. We assessed heterogeneity by including an interaction term between the variable used for stratification and the biomarker of interest.

We conducted a sensitivity analysis excluding women using exogenous hormones at inclusion (*n* = 86 women, leading to exclusion of 74 case–control pairs).

All statistical tests were two-sided. All analyses were performed using R Studio Version 1.2.5042 and R version 4.0.3.

## Results

On average, cases were aged 38.8 at inclusion (38.7 in controls) and in the 298 cases with available immunohistochemistry data, 72.1% of tumors were ER-positive, 68.8% were PR-positive, and 14.1% were triple-negative (Table 1). Characteristics of the cases by hormone receptor status are shown in Supplementary Table 1 (see Additional File 1).

**Table 1:** Main characteristics of study population by case/control status

	Controls (*n* = 453)	Cases (*n* = 453)
	Mean (SD) or N (%)	Mean (SD) or N (%)
Age at inclusion (years)	38.67 (5.18)	38.76 (5.08)
Age at menarche (years)	12.52 (1.83)	12.47 (1.67)
Age at first full-term pregnancy (years)^a^	21.89 (5.16)	23.91 (5.86)
Number of full-term pregnancies (%)		
0	34 (7.5)	82 (18.1)
1	97 (21.4)	129 (28.5)
2	189 (41.7)	156 (34.4)
3 or more	133 (29.4)	86 (19.0)
Total duration of breastfeeding in parous women (months) (%)^a^		
< 8	109 (26.0)	155 (41.8)
8–22	143 (34.1)	120 (32.3)
> 22	167 (39.9)	96 (25.9)
Use of hormones at blood collection (%)	48 (10.6)	38 (8.4)
Personal history of benign breast disease (%)	55 (12.1)	165 (36.4)
Level of education		
≤ Primary school	95 (21.0)	58 (12.8)
Secondary school	247 (54.5)	227 (50.1)
> Secondary school	111 (24.5)	168 (37.1)
Never smoker (%)	203 (44.8)	239 (52.8)
Moderate physical activity (hours/day)	3.2 (2.6)	2.4 (2.6)
Body mass index (kg/m^2^)	29.2 (9.7)	26.4 (5.0)
< 25 kg/m^2^ (%)	119 (26.3)	199 (43.9)
25–29.9 kg/m^2^ (%)	175 (38.6)	175 (38.6)
≥ 30 kg/m^2^ (%)	159 (35.1)	79 (17.4)
Waist circumference (cm)	94.5 (15.1)	91.0 (12.3)
Hip circumference (cm)	106.5 (12.9)	103.5 (9.9)
Waist/hip ratio	0.89 (0.10)	0.88 (0.08)
Tumor characteristics^b^		
ER-positive (irrespective of other receptors)		215 (72.1)
PR-positive (irrespective of other receptors)		205 (68.8)
HER2-positive (irrespective of other receptors)		50 (16.8)
Triple Negative: ER-/PR-/HER2-		64 (14.1)
**Biomarkers (geometric mean (geometric SD))**		
IL-6 (pg/ml)	0.69 (1.92)	0.73 (2.29)
IL-8 (pg/ml) ^c^	7.74 (2.05)	8.27 (2.04)
IL-10 (pg/ml)	0.19 (2.27)	0.18 (2.05)
TNF-α (pg/ml)	1.86 (1.50)	1.90 (1.56)
IFN-γ (pg/ml)	4.44 (2.32)	4.07 (2.34)
Leptin (ng/ml) ^d^	14.44 (2.48)	11.19 (2.51)
Adiponectin (µg/ml)	8.12 (4.47)	8.52 (4.21)
Leptin/adiponectin ratio	1.74 (5.72)	1.29 (5.85)

*Abbreviations: ER* Estrogen receptor, *HER2* Human epidermal growth factor receptor 2, *IFN-γ* Interferon γ, *IL-6* Interleukin 6, *IL-8* Interleukin 8, *IL-10* Interleukin 10, *PR* Progesterone receptor, *SD* Standard deviation, *TNF-α* Tumor necrosis factor α.

^a^In parous women only

^b^Available on 298 (65.8%) cases

^c^Available on 314 cases and 314 controls

^d^Available on 430 cases and 430 controls

Compared with controls, cases were more likely to be nulliparous (18.1% versus 7.5%). Parous cases were on average older at their first full-term pregnancy (23.9 years versus 21.9 years) and breastfed less (41.8% did not breastfed or breastfed less than 8 months (first tertile) versus 26.0%) than parous controls. Cases had more frequently a history of benign breast disease (36.4% versus 12.1%) and a level of education above secondary school (37.1% versus 24.5%) than controls. Compared with controls, cases had a lower BMI (26.4 kg/m^2^ versus 29.2 kg/m^2^), waist circumference (91.0 cm versus 94.5 cm), and hip circumference (103.5 cm versus 106.5 cm).

**Fig. 1:**
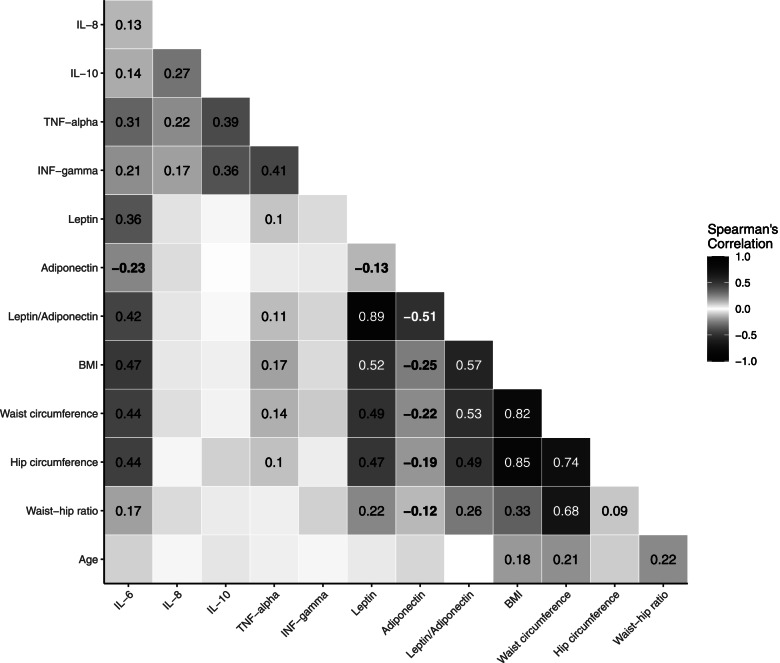
Age-adjusted Spearman correlation coefficients between concentrations* of inflammation markers and anthropometric measures among control participants. *Concentration of biomarkers as residual of log-transformed variables regressed on analytical batch. Only statistically significant correlations (*P*-value < 0.05) are shown. Bold indicate negative correlation coefficients. Abbreviations: BMI body mass index; IFN interferon, IL interleukin; OR odds ratio; SD standard deviation; TNF tumor necrosis factor

Age-adjusted Spearman correlation coefficients among control participants were moderate between circulating levels of different cytokines (IL-6, IL-8, IL-10), TNF-α, and IFN-γ (0.13 ≤ r ≤ 0.41) (Fig. 1). In addition, IL-6 was positively correlated with leptin (*r *= 0.36) and the leptin/adiponectin ratio (*r *= 0.42), negatively correlated with adiponectin (*r* = -0.23), and showed stronger correlations with anthropometric measures than with biomarkers (0.47 with BMI, 0.44 with waist circumference, 0.44 with hip circumference). Leptin showed the strongest positive correlations with BMI, waist and hip circumferences (respectively 0.52, 0.49, and 0.47) and leptin/adiponectin ratio (respectively 0.57, 0.53, and 0.49), while adiponectin showed negative correlations with BMI, waist and hip circumferences (respectively -0.25, -0.22, and -0.19). Correlations between waist-hip ratio and biomarkers were lower than with other anthropometric measures (0.12 ≤|r|≤ 0.22). Weak correlations were also seen between TNF-α and anthropometric measures (0.10 to 0.17).

In the model including only matching factors (Table 2), no significant associations were observed for interleukins, TNF-α, and IFN-γ with breast cancer risk, while leptin and leptin/adiponectin ratio showed significant negative associations (leptin: OR_per SD increment_ (95% CI) = 0.76 (0.66–0.87), OR_Q4vsQ1_ = 0.48 (0.33–0.70), *P*-trend < 0.001; leptin/adiponectin ratio: OR_per SD increment_ = 0.75 (0.65–0.86), OR_Q4vsQ1_ = 0.53 (0.36–0.79), *P*-trend < 0.001). A positive but borderline statistically significant association was also observed for adiponectin (OR_per SD increment_ = 1.14 (0.99–1.31); OR_Q4vsQ1_ = 1.45 (0.98–2.14), *P*-trend = 0.04).

**Table 2:** Associations between inflammatory biomarkers and breast cancer risk in premenopausal women from the PRECAMA study

	Cases/controls	Univariate^a^		BMI-adjusted^b^		WC-adjusted^c^		Fully-adjusted^d^	
		OR (95% CI)	*P-value*	OR (95% CI)	*P-value*	OR (95% CI)	*P-value*	OR (95% CI)	*P-value*
**IL-6, per SD increase**	453/453	1.07 (0.94–1.22)	*0.29*	1.32 (1.12–1.55)	*0.001*	1.24 (1.07–1.44)	*0.005*	1.33 (1.11–1.60)	*0.002*
Quartile 1	140/114	1 (ref.)		1 (ref.)		1 (ref.)		1 (ref.)	
Quartile 2	88/113	0.64 (0.44–0.94)		0.78 (0.52–1.16)		0.74 (0.50–1.09)		0.70 (0.43–1.13)	
Quartile 3	88/113	0.64 (0.44–0.93)		0.90 (0.60–1.35)		0.80 (0.54–1.19)		0.83 (0.51–1.35)	
Quartile 4	137/113	0.96 (0.67–1.36)		1.55 (1.04–2.30)		1.32 (0.90–1.95)		1.36 (0.85–2.18)	
		P-trend	*0.87*	P-trend	*0.01*	P-trend	*0.06*	P-trend	*0.07*
**IL-8, per SD increase**	314/314	1.11 (0.94–1.3)	*0.21*	1.15 (0.97–1.36)	*0.11*	1.15 (0.98–1.36)	*0.09*	1.15 (0.93–1.44)	*0.20*
Quartile 1	67/79	1 (ref.)		1 (ref.)		1 (ref.)		1 (ref.)	
Quartile 2	84/78	1.31 (0.82–2.09)		1.3 (0.79–2.13)		1.35 (0.84–2.18)		1.99 (1.04–3.78)	
Quartile 3	63/78	0.96 (0.60–1.55)		0.98 (0.60–1.63)		1.04 (0.64–1.70)		1.17 (0.60–2.26)	
Quartile 4	100/79	1.49 (0.95–2.32)		1.54 (0.96–2.48)		1.59 (1.01–2.52)		2.16 (1.14–4.07)	
		P-trend	*0.13*	P-trend	*0.10*	P-trend	*0.07*	P-trend	*0.06*
**IL-10, per SD increase**	453/453	0.92 (0.81–1.05)	*0.22*	0.93 (0.80–1.07)	*0.28*	0.94 (0.82–1.08)	*0.37*	0.95 (0.79–1.13)	*0.54*
Quartile 1	124/114	1 (ref.)		1 (ref.)		1 (ref.)		1 (ref.)	
Quartile 2	100/114	0.79 (0.54–1.16)		0.77 (0.52–1.15)		0.80 (0.54–1.18)		0.80 (0.50–1.29)	
Quartile 3	129/112	1.04 (0.73–1.49)		1.01 (0.69–1.47)		1.04 (0.72–1.49)		1.09 (0.70–1.70)	
Quartile 4	100/113	0.80 (0.55–1.17)		0.77 (0.51–1.14)		0.81 (0.55–1.19)		0.76 (0.47–1.24)	
		P-trend	*0.46*	P-trend	*0.36*	P-trend	*0.50*	P-trend	*0.44*
**TNF-α, per SD increase**	453/453	1.06 (0.93–1.21)	*0.38*	1.14 (0.99–1.32)	*0.07*	1.14 (0.99–1.32)	*0.07*	1.32 (1.11–1.58)	*0.002*
Quartile 1	119/114	1 (ref.)		1 (ref.)		1 (ref.)		1 (ref.)	
Quartile 2	108/113	0.91 (0.62–1.32)		1.01 (0.68–1.49)		0.99 (0.68–1.45)		1.55 (0.96–2.49)	
Quartile 3	107/113	0.90 (0.61–1.32)		0.95 (0.63–1.41)		0.96 (0.65–1.42)		1.49 (0.90–2.47)	
Quartile 4	119/113	1.01 (0.70–1.45)		1.32 (0.89–1.94)		1.25 (0.85–1.83)		2.03 (1.26–3.26)	
		P-trend	*0.96*	P-trend	*0.20*	P-trend	*0.28*	P-trend	*0.006*
**IFN-γ, per SD increase**	453/453	0.90 (0.80–1.03)	*0.12*	0.93 (0.82–1.07)	*0.32*	0.94 (0.82–1.07)	*0.33*	0.94 (0.80–1.09)	*0.42*
Quartile 1	135/114	1 (ref.)		1 (ref.)		1 (ref.)		1 (ref.)	
Quartile 2	118/113	0.90 (0.64–1.27)		0.92 (0.64–1.33)		0.9 (0.63–1.28)		1.35 (0.86–2.12)	
Quartile 3	96/113	0.74 (0.52–1.06)		0.79 (0.55–1.14)		0.77 (0.54–1.10)		0.91 (0.58–1.42)	
Quartile 4	104/113	0.80 (0.57–1.14)		0.87 (0.60–1.25)		0.86 (0.60–1.24)		0.87 (0.56–1.35)	
		P-trend	*0.14*	P-trend	*0.34*	P-trend	*0.32*	P-trend	*0.29*
**Leptin, per SD increase**	430/430	0.76 (0.66–0.87)	< *0.001*	0.94 (0.80–1.1)	*0.43*	0.84 (0.72–0.98)	*0.03*	0.82 (0.67–1.00)	*0.05*
Quartile 1	162/108	1 (ref.)		1 (ref.)		1 (ref.)		1 (ref.)	
Quartile 2	112/107	0.72 (0.50–1.03)		0.87 (0.60–1.28)		0.78 (0.54–1.13)		0.77 (0.48–1.22)	
Quartile 3	81/107	0.52 (0.36–0.76)		0.69 (0.46–1.03)		0.59 (0.40–0.88)		0.55 (0.34–0.89)	
Quartile 4	75/108	0.48 (0.33–0.70)		0.84 (0.54–1.32)		0.62 (0.41–0.96)		0.62 (0.36–1.08)	
		P-trend	< *0.001*	P-trend	*0.21*	P-trend	*0.008*	P-trend	*0.03*
**Adiponectin, per SD increase**	453/453	1.14 (0.99–1.31)	*0.08*	1.02 (0.88–1.19)	*0.78*	1.07 (0.92–1.24)	*0.40*	1.05 (0.87–1.27)	*0.59*
Quartile 1	99/114	1 (ref.)		1 (ref.)		1 (ref.)		1 (ref.)	
Quartile 2	99/113	1.01 (0.69–1.48)		0.93 (0.62–1.39)		0.96 (0.65–1.43)		1.17 (0.72–1.91)	
Quartile 3	119/113	1.25 (0.86–1.80)		1.01 (0.68–1.49)		1.11 (0.76–1.62)		1.00 (0.62–1.62)	
Quartile 4	136/113	1.45 (0.98–2.14)		1.16 (0.77–1.74)		1.25 (0.83–1.86)		1.30 (0.79–2.13)	
		P-trend	*0.04*	P-trend	*0.43*	P-trend	*0.23*	P-trend	*0.38*
**Leptin/adiponectin, per SD increase**	430/430	0.75 (0.65–0.86)	< *0.001*	0.93 (0.79–1.11)	*0.43*	0.83 (0.71–0.98)	*0.02*	0.81 (0.66–1.00)	*0.05*
Quartile 1	146/108	1 (ref.)		1 (ref.)		1 (ref.)		1 (ref.)	
Quartile 2	126/107	0.89 (0.61–1.28)		1.06 (0.72–1.56)		0.98 (0.67–1.43)		0.90 (0.56–1.44)	
Quartile 3	81/107	0.56 (0.38–0.83)		0.78 (0.52–1.18)		0.66 (0.44–1.00)		0.62 (0.38–1.01)	
Quartile 4	77/108	0.53 (0.36–0.79)		0.98 (0.62–1.55)		0.72 (0.47–1.13)		0.74 (0.42–1.31)	
		P-trend	< *0.001*	P-trend	*0.62*	P-trend	*0.07*	P-trend	*0.13*

^a^ Matched on age (± 3 years), city district of residence, and health insurance institution

^b^ Univariate model, additionally adjusted for BMI (continuous)

^c^ Univariate model, additionally adjusted for WC (continuous)

^d^ Univariate model, additionally adjusted for BMI (continuous), age at menarche (years, continuous), number of full-term pregnancies (0/1/2/ ≥ 3), age at first pregnancy (nulliparous, tertiles), breastfeeding duration (nulliparous, tertiles), use of hormones at blood collection (yes/no), personal history of benign breast disease (yes/no), family history of breast cancer in first-degree relatives (yes/no), smoking status (ever smoker/never smoker), alcohol consumption (g/day), moderate physical activity (hours/day), education level (up to primary school/secondary school/longer than secondary school), and adult height (continuous)

*Abbreviations: BMI* Body mass index, *CI* Confidence interval, *IFN-γ* Interferon-γ, *IL* Interleukin, *OR* Odds ratio, *SD* Standard deviation, *TNF-α* Tumor necrosis factor α, *WC* Waist circumference

After adjustment for BMI, associations between leptin, adiponectin and leptin/adiponectin ratio and breast cancer risk were no longer significant, while a significant positive association was observed for IL-6 (OR_per SD increment_ = 1.32 (1.12–1.55); OR_Q4vsQ1_ = 1.55 (1.04–2.30), *P*-trend = 0.01).

When adjusting the univariate model for waist circumference, instead of BMI, the association of IL-6 and breast cancer risk was attenuated (OR_per SD increment_ = 1.24 (1.07–1.44); OR_Q4vsQ1_ = 1.32 (0.90–1.95), *P*-trend = 0.06). In the same waist circumference-adjusted model, IL-8 was positively associated with risk (OR_per SD increment_ = 1.15 (0.98–1.36); OR_Q4vsQ1_ = 1.59 (1.01–2.52), *P*-trend = 0.07). Leptin and leptin/adiponectin ratio were negatively associated with breast cancer risk when adjusting for waist circumference, although associations were attenuated as compared with the univariate model.

In the fully adjusted model (which included BMI but not waist circumference), IL-6 was positively associated with breast cancer risk (continuous variable only, OR_per SD increment_ = 1.33 (1.11–1.60)), as was TNF-α (OR_per SD increment_ = 1.32 (1.11–1.58); OR_Q4vsQ1_ = 2.03 (1.26–3.26), *P*-trend = 0.006). A significant positive association was observed for IL-8 in quartiles (OR_Q4vsQ1_ = 2.16 (1.14–4.07), *P*-trend = 0.06), but not for the continuous variable (OR_per SD increment_ = 1.15 (0.93–1.44)). Borderline negative associations were observed for leptin (OR_per SD increment_ = 0.82 (0.67–1.00)) and leptin/adiponectin ratio (OR_per SD increment_ = 0.81 (0.66–1.00)). No associations were observed overall for IL-10 and IFN-γ.

Although no departure from linearity was suggested from the linear trend test across quartiles, natural cubic spline models indicated evidence for non-linearity for IL-6 (*P*-value = 0.03) and TNF-alpha (*P*-value = 0.04) (Supplementary Fig. 1, see Additional File 1), but not for other biomarkers (all *P*-values ≥ 0.30).

**Fig. 2:**
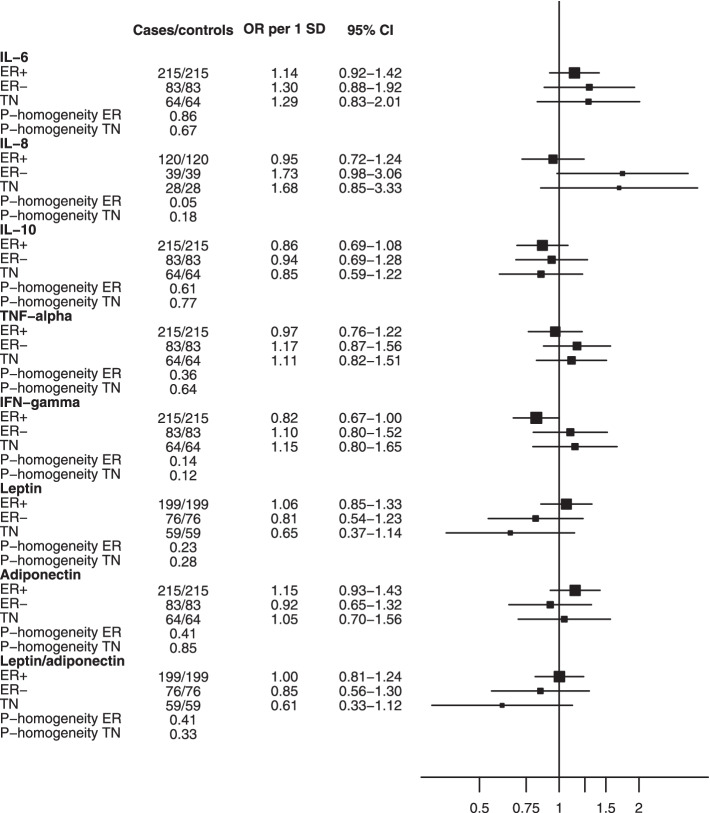
Associations between inflammatory biomarkers and breast cancer, by estrogen receptor status and in triple-negative tumors. Odds ratios are per standard deviation increase in residuals of log-transformed biomarker concentration regressed on analytical batch, estimated from conditional logistic regression models adjusted for BMI. P-homogeneity ER compares estrogen receptor negative and positive tumors. P-homogeneity TN compares triple-negative and non-triple-negative tumors. Abbreviations: *BMI* body mass index, *CI* confidence interval, *ER* + estrogen receptor positive, *ER*- estrogen receptor negative, *IFN* interferon, *IL* interleukin, *OR* odds ratio, *SD* standard deviation, *TN* triple negative, *TNF* tumor necrosis factor

When stratifying analyses by tumor immunohistochemistry (Fig. 2), IL-8 was observed to be positively associated with breast cancer risk for ER-negative tumors only (ER-positive: OR_per SD increment_ = 0.95 (0.72–1.24); ER-negative: OR_per SD increment_ = 1.73 (0.98–3.06); P-homogeneity_ER+ vs ER-_ = 0.05). In contrast, a negative association was observed for IFN-γ in ER + tumors only (OR_per SD increment_ = 0.82 (0.67–1.00), although the test for heterogeneity for receptor status was not significant (P-homogeneity_ER+ vs ER-_ = 0.14). No evidence of heterogeneity was observed for other biomarkers (all P-homogeneity > 0.23) or when comparing triple-negative to non-triple-negative tumors (all P-homogeneity > 0.12).

**Fig. 3:**
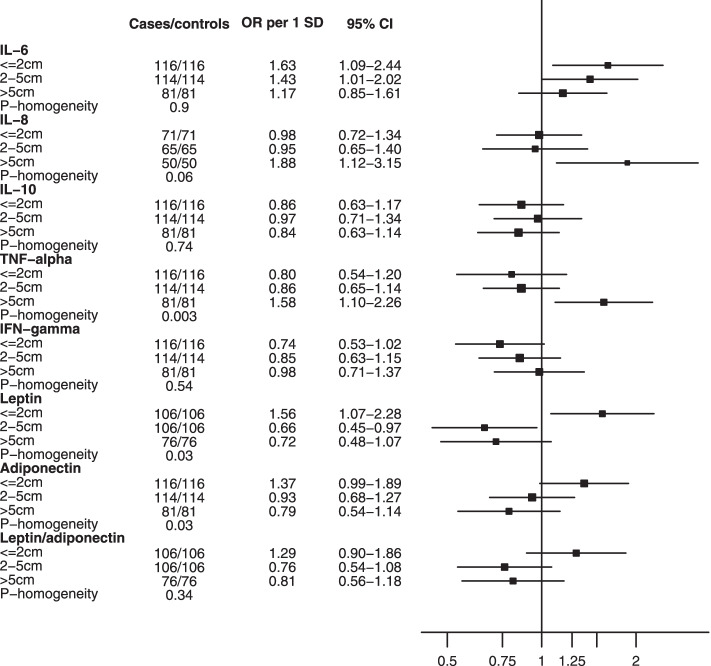
Associations between inflammatory biomarkers and breast cancer risk, by tumor size. Odds ratios are per standard deviation increase in residuals of log-transformed biomarker concentration regressed on analytical batch, estimated from conditional logistic regression models adjusted for BMI. Abbreviations: *BMI* Body mass index, *CI* Confidence interval, *IFN* Interferon, *IL* Interleukin, *OR* Odds ratio, *SD* Standard deviation, *TN* Triple negative, *TNF* Tumor necrosis factor.

When exploring associations by tumor size, little evidence of heterogeneity was observed for IL-6 and breast cancer risk (Fig. 3, *P*-homogeneity = 0.90), although significant associations were observed only in tumors <  = 2 cm and 2–5 cm (< = 2 cm: OR_per SD increment_ = 1.63 (1.09–2.44); 2-5 cm: OR_per SD increment_ = 1.43 (1.01–2.02) but not for larger tumors (> 5 cm: OR_per SD increment_ = 1.17 (0.85–1.61)).

For IL-8 and TNF-α, a significant positive association was observed only for the largest tumors (IL-8: OR_per SD increment_ = 1.88 (1.12–3.15), P-homogeneity = 0.06; TNF-α: OR_per SD increment_ = 1.58 (1.10–2.26), P-homogeneity = 0.003). Leptin and adiponectin were positively associated with risk in smaller tumors (leptin: OR_per SD increment_ = 1.56 (1.07–2.28); adiponectin: OR_per SD increment_ = 1.37 (0.99–1.89)), with P-homogeneity = 0.03 for both biomarkers), while negative associations were observed for larger tumors, only significant for leptin in tumors of 2 to 5 cm (OR_per SD increment_ = 0.66 (0.45–0.97)).

When stratifying analyses on BMI using 25 kg/m^2^ as a cut-off, no interaction between BMI and any of the biomarkers was observed (all *P*-interaction > 0.40, not shown). However, when using a cut-off of 30 kg/m^2^, a significant interaction with IL-8 (*P*-interaction = 0.03; < 30 kg/m^2^: OR_per SD increment_ = 1.06 (0.88–1.28); ≥ 30 kg/m^2^: OR_per SD increment_ = 1.49 (1.05–2.15)), and a borderline significant interaction with TNF-α (P-interaction = 0.05; < 30 kg/m^2^: OR_per SD increment_ = 1.08 (0.93–1.26); ≥ 30 kg/m^2^: OR_per SD increment_ = 1.42 (1.05–1.95)) were observed. When restricting the analysis to women not using exogenous hormones, associations remained unchanged, except for a positive association between IL-8 and breast cancer risk overall in all multivariate models (BMI-adjusted: OR_per SD increment_ = 1.22 (1.01–1.48); WC-adjusted: OR_per SD increment_ = 1.22 (1.02–1.46); fully adjusted: OR_per SD increment_ = 1.46 (1.13–1.90), not tabulated).

## Discussion

This analysis of seven inflammatory biomarkers and breast cancer risk in a case–control study in premenopausal women from Latin America showed that IL-6 and TNF-α were positively associated with breast cancer risk when accounting for adiposity and other breast cancer risk factors. While the association with IL-6 was consistent across different breast cancer subtypes and tumor sizes, the association with TNF-α was limited to larger tumors. A positive association was observed for IL-8 in ER-negative and in larger tumors, and in hormones non-users at blood donation, while a negative association was observed for IFN-γ in ER-positive tumors only. Leptin and leptin/adiponectin ratio showed negative associations with breast cancer risk in univariate models, that were attenuated when adiposity was accounted for. When associations were explored by tumor size, however, negative associations were observed in larger tumors (> 2 cm) and positive associations in smaller tumors.

The positive association between IL-6 and breast cancer risk is in line with the only prospective study of biomarkers of chronic inflammation and risk of premenopausal breast cancer we identified [16]. In this study among Italian women, a positive association was reported between circulating IL-6 levels and breast cancer risk independently from overall adiposity. In addition, several cross-sectional studies [22–24] have shown higher circulating IL-6 concentrations in breast cancer patients than in controls, as often reported for cancer patients overall [25, 26]. However, menopausal status of these women was not specified in these studies, and adjustment for obesity was not considered. As well, these studies reported increasing IL-6 concentrations with increasing disease stage [22–24]. However, in the present work, IL-6 associations with breast cancer risk did not vary by tumor size. Mechanistic studies suggest a complex role of IL-6 in cancer development, with effects at both local and systemic levels, mainly mediated through the JAK/STAT3 signaling pathways [26, 27]. Stimulation of the IL-6 / JAK / STAT3 pathway in cancer cells modulates the expression of several genes involved in the proliferation, survival and transformation of tumor cells [27]. Activation of this pathway is also suggested to decrease anti-tumor immunity by creating an immunosuppressive tumor microenvironment [27].

IL-8 concentrations were previously reported to be higher in breast cancer cases than in healthy women [24, 28], with higher concentrations in ER-negative than ER-positive tumors [24], defining IL-8 as a possible marker of ER-negative and/or HER2-positive breast cancers [29]. This may rely on a close crosstalk between IL-8 and ER expression in breast tissue [29, 30]. However, it has been shown that IL-8 can increase invasiveness of breast cancer cells, irrespective of their ER status [29], for instance by promoting angiogenesis [31]. It has also been shown that highly metastatic cell lines produce more IL-8 than lower metastatic cell lines [32], which may support the association we reported in this study showing an association only for the largest tumors. This is also in line with previous studies reporting increased concentrations of IL-8 among patients with metastatic disease [28]. The observation of a positive association only when hormone users were excluded could result from a potential influence of oral contraceptive use on inflammatory markers, although not specific to IL-8 [33]. Another possible explanation is that oral contraceptive users are at increased risk for breast cancer [34], and this association may mask the association with IL-8.

IL-10 is known to be an anti-inflammatory cytokine reported to have mostly anti-tumor properties, based on experimental and clinical data, but pro-tumor actions have sometimes been reported based on in vitro and animal studies [35]. Higher levels of serum IL-10 have also been reported in breast cancer patients as compared to controls [23], but we did not detect any association in this work, for all cancers, nor by receptor expression, or by tumor size.

Our finding of a positive association between TNF-α and breast cancer is consistent with several case–control studies showing higher serum TNF-α concentrations in breast cancer patient than controls [24, 36–39]. These studies however were not focused on premenopausal women only. Furthermore, concentrations of TNF-α have been seen to correlate with several disease characteristics [39]. One study [24] indicated that the difference in TNF-α concentrations between breast cancer patients and controls was significant only for stage III cancers, consistent with the positive association that we observed only for large tumors. The only prospective study analyzing the association between TNF-α and breast cancer risk in premenopausal women showed a positive association across tertiles of TNF-α (*P*-trend = 0.02) [16]. This pro-inflammatory cytokine is suspected to be involved in tumor progression and metastasis but its role in breast cancer remains challenging to understand, as results from studies on breast cancer-derived cell lines indicate that TNF-α may actually induce either apoptosis or cell proliferation, depending on the cellular context [39, 40].

IFN-γ has anti-tumor properties, i.e., pro-apoptotic, anti-angiogenic, and promotor of anti-tumor immune response [41], that are in line with the negative association we observed in our study. The few studies we identified exploring the association between IFN-γ and breast cancer risk in breast cancer patients versus controls (unspecified menopausal status) suggested either no difference [42] (250 aged-matched case–control pairs) or lower concentrations in cases [43] (29 controls, 55 patients not receiving chemotherapy, 32 receiving chemotherapy).

It is not clear why in our study we observed a significant association in ER-positive tumors only. However, since the heterogeneity between tumors with different estrogen receptors was not statistically significant, the observed difference in association could result from a lack of statistical power in the ER-negative subgroup.

In the present work, leptin was inversely associated with breast cancer risk in both univariate and fully adjusted model, however this association lost significance when the univariate model was adjusted for BMI. Leptin is a pro-inflammatory adipokine [44] strongly positively associated with BMI. Since we reported a strong inverse association between BMI and breast cancer in this population [6], the negative association between leptin and breast cancer may result from residual confounding. However, our results are consistent with an Italian prospective study [16] reporting a negative association in premenopausal women, even when BMI was considered. As well, two additional prospective studies [45, 46] reported negative associations, although they did not reach statistical significance in the fully adjusted model. When stratifying analysis by tumor stage or in situ versus invasive, the latter studies [45, 46] reported negative associations in the less advanced tumors, which contrasts with our observation of a positive association in the smallest tumors. In line with results on leptin, leptin/adiponectin ratio, a suggested marker of insulin resistance [47], was also negatively associated with breast cancer risk in the present work.

We did not observe any association of adiponectin with breast cancer risk, consistent with findings from three prospective studies in premenopausal women [16, 45, 48] and two case–control studies [49]. Baseline data from a randomized controlled trial, in which premenopausal women with intraepithelial neoplasia or micro-invasive breast cancer were compared to healthy high-risk women (5-year Gail score > 1.3%), showed a decrease in risk with increasing adiponectin (but not leptin) concentrations [50]. Similar to what we observed for leptin, a borderline positive association with breast cancer risk was observed for small tumors only, which contrasts with the known anti-inflammatory properties of this adipokine [44].

Strengths of PRECAMA, the largest ongoing multicentric population-based case–control study of premenopausal breast cancer in Latin America, include the use of standardized protocols for collection of a wide range of lifestyle and anthropometric factors. We were therefore able to account for adiposity, which is strongly associated with breast cancer risk in this population [6], and other lifestyle variables, even if residual confounding cannot be entirely ruled out. The availability of centralized immunohistochemistry analyses reduced inter-laboratory variability and enabled us to stratify our analysis by breast cancer subtype. In addition, the use of a highly sensitive and specific method for measuring circulating biomarkers allowed us to measure all the cytokines of interest, even if present at very low concentrations, in all the samples in the study.

A major limitation is the case–control design of this study, that does not enable to draw any conclusion regarding the temporality of the observed associations or their potential causality. However, cases were recruited, and biological samples collected, before the start of any treatment, and tumor size was carefully recorded, which allowed us to analyze the associations accounting for disease advancement. Another limitation is that sample size was sometimes limited for subgroup analysis and resulted in limited statistical power. In addition, a total of 7 biomarkers and one ratio were analyzed overall and in stratified analyses without adjustment for multiple tests, which increases the risk of chance findings and warrants caution in the interpretation of our findings. Lastly, the generalization of these results to other age groups or geographical regions requires careful consideration.

## Conclusions

In conclusion, this work showed that IL-6 and TNF-α are positively associated with breast cancer in premenopausal women in Latin America. While associations did not vary by hormone receptor status, except for IL-8, heterogeneity by tumor size was observed for several biomarkers (IL-8, TNF-α, leptin, adiponectin). These findings add useful information for the characterization of the obesity and metabolic health and breast cancer in premenopausal women. However, given the complex interplay between biomarkers of inflammation and cancer development, largest studies of prospective design are needed to confirm these findings in premenopausal women.

## Disclaimer

Where authors are identified as personnel of the International Agency for Research on Cancer/ World Health Organization, the authors alone are responsible for the views expressed in this article and they do not necessarily represent the decisions, policy or views of the International Agency for Research on Cancer/ World Health Organization.

## Supplementary Information

**Additional file 1.** Main characteristics of cases by hormone receptor status, and for triple-negative tumors (Supplementary Table 1). Associations between IL-6 and TNF-alpha and breast cancer risk allowing for non-linear effects (natural cubic splines) (Supplementary Figure 1); Abbreviations: BMI body mass index; ER Estrogen receptor; HER2 human epidermal growth factor receptor 2; IFN-γ interferon γ; IL-6 interleukin 6; IL-8 interleukin 8; IL-10 interleukin 10; OR odds ratio; PR progesterone receptor; SD standard deviation; TNF-α tumor necrosis factor α. (DOC 109 KB).

## Data Availability

The data that support the findings of this study are available from the PRECAMA centres that provided them, but restrictions apply to the availability of these data, which were used under license for the current study, and so are not publicly available. Data are however available from the authors upon reasonable request and with permission of the PRECAMA Steering Committee.

## Abbreviations

BMI: Body mass index
CI: Confidence interval
ER: Estrogen receptor
HER2: Human epidermal growth factor receptor 2
IARC: International Agency for Research on Cancer
IFN-γ: Interferon γ
IL-6: Interleukin 6
IL-8: Interleukin 8
IL-10: Interleukin 10
OR: Odds ratio
PR: Progesterone receptor
SD: Standard deviation
TN: Triple negative
TNF-α: Tumor necrosis factor
