# Supplementary material for: Inflammatory biomarkers and risk of breast cancer among young women in Latin America: a case-control study
Source: BMC Cancer. 2022 Aug 11;22:877. doi: 10.1186/s12885-022-09975-6 (PMC9367082; doi:10.1186/s12885-022-09975-6)
Supplement: Supplementary file 1 — Additional file 1. Main characteristics of cases by hormone receptor status, and for triple-negative tumors (Supplementary Table 1). Associations between IL-6 and TNF-alpha and breast cancer risk allowing for non-linear effects (natural cubic splines) (Supplementary Figure 1); Abbreviations: BMI body mass index; ER Estrogen receptor; HER2 human epidermal growth factor receptor 2; IFN-γ interferon γ; IL-6 interleukin 6; IL-8 interleukin 8; IL-10 interleukin 10; OR odds ratio; PR progesterone receptor; SD standard deviation; TNF-α tumor necrosis factor α. (DOC 109 KB). [file 12885_2022_9975_MOESM1_ESM.docx]

**Supplementary Figure 1. Associations between IL-6 and TNF-alpha and breast cancer risk allowing for non-linear effects (natural cubic splines)**

Biomarkers were modelled as standardized residuals on analytical batch and models were adjusted for BMI. Biomarkers concentrations displayed on the X-axis correspond to normalized concentrations (after removing batch effect).

Abbreviations: BMI body mass index; IL interleukin; OR odds ratio; TNF tumor necrosis factor.


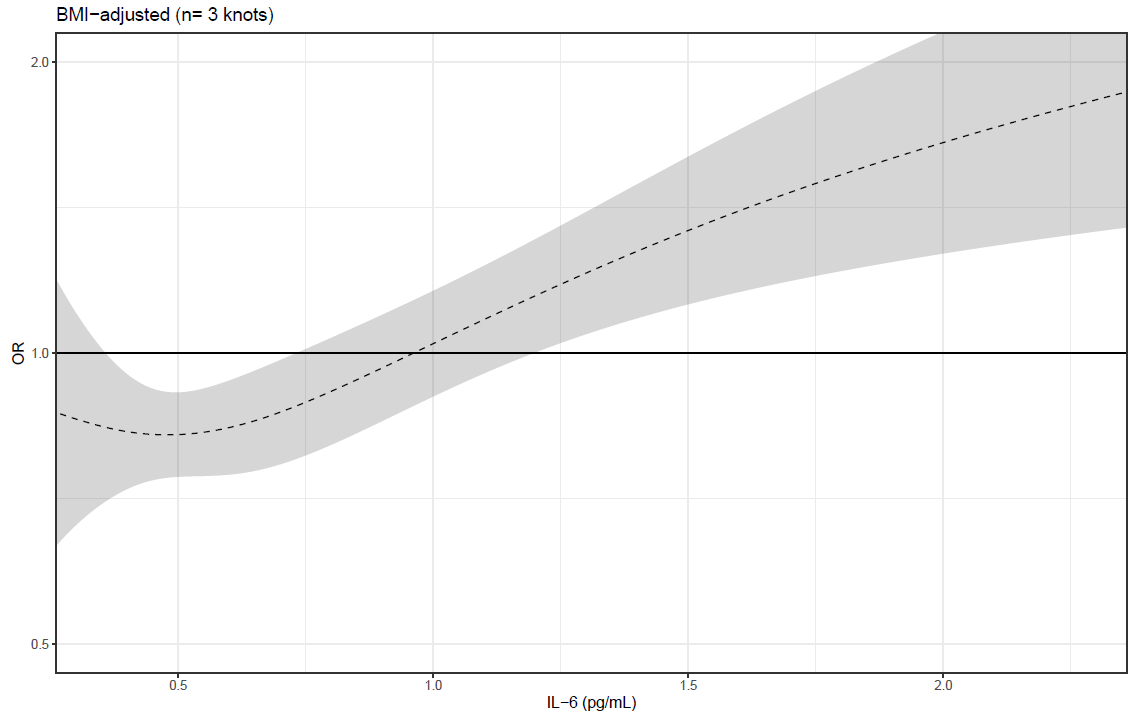


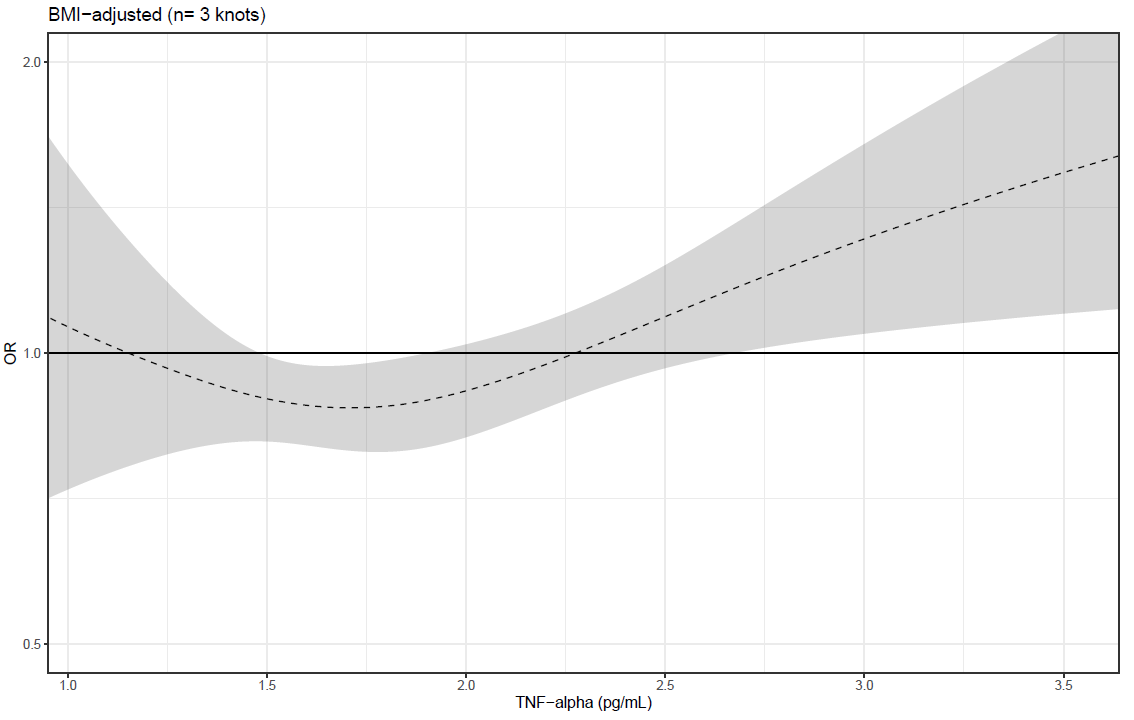


**Supplementary Table 1. Main characteristics of cases by hormone receptor status, and for triple-negative tumors**

|  | **ER-positive**  **(n=215)** | **PR-positive**  **(n=205)** | **HER2-positive**  **(n=50)** | **Triple-negative**  **(n=64)** |
| --- | --- | --- | --- | --- |
|  | Mean (SD) or N (%) | Mean (SD) or N (%) | Mean (SD) or N (%) | Mean (SD) or N (%) |
| Age at inclusion (years) | 39.03 (5.26) | 39.15 (5.25) | 37.94 (5.53) | 37.66 (5.07) |
| Age at menarche (years) | 12.55 (1.53) | 12.55 (1.55) | 12.68 (1.60) | 11.97 (2.02) |
| Age at first full-term pregnancy (years)^a^ | 24.58 (5.83) | 24.64 (5.91) | 25.45 (6.75) | 21.04 (4.86) |
| Number of full-term pregnancies (%) |  |  |  |  |
| 0 | 39 (18.1) | 38 (18.5) | 8 (16.0) | 9 (14.1) |
| 1 | 63 (29.3) | 60 (29.3) | 20 (40.0) | 15 (23.4) |
| 2 | 75 (34.9) | 68 (33.2) | 13 (26.0) | 26 (40.6) |
| 3 or more | 38 (17.7) | 39 (19.0) | 9 (18.0) | 14 (21.9) |
| Total duration of breastfeeding in parous women (months) (%)^a^ |  |  |  |  |
| <8 | 73 (41.5) | 67 (40.1) | 17 (40.5) | 18 (32.7) |
| 8-22 | 58 (33.0) | 57 (34.1) | 14 (33.3) | 20 (36.4) |
| >22 | 45 (25.5) | 43 (25.7) | 11 (26.2) | 17 (30.9) |
| Use of hormones at blood collection (%) | 22 (10.2) | 22 (10.7) | 6 (12.0) | 6 (9.4) |
| Personal history of benign breast disease (%) | 84 (39.1) | 82 (40.0) | 19 (38.0) | 21 (32.8) |
| Level of education |  |  |  |  |
| ≤ Primary school | 26 (12.1) | 26 (12.7) | 8 (16.0) | 16 (25.0) |
| Secondary school | 112 (52.1) | 106 (51.7) | 28 (56.0) | 30 (46.9) |
| > Secondary school | 77 (35.8) | 73 (35.6) | 14 (28.0) | 18 (28.1) |
| Never smoker (%) | 114 (53.0) | 107 (52.2) | 29 (58.0) | 37 (57.8) |
| Moderate physical activity (hours/day) | 2.6 (2.8) | 2.7 (2.8) | 2.00 (2.1) | 2.7 (2.6) |
| Body mass index (kg/m²) | 26.4 (4.9) | 26.4 (4.9) | 25.4 (5.1) | 26.9 (4.8) |
| <25 kg/m² (%) | 91 (42.3) | 85 (41.5) | 27 (54.0) | 26 (40.6) |
| 25-29.9 kg/m² (%) | 90 (41.9) | 88 (42.9) | 17 (34.0) | 24 (37.5) |
| ≥30 kg/m² (%) | 34 (15.8) | 32 (15.6) | 6 (12.0) | 14 (21.9) |
| Waist circumference (cm) | 89.8 (12.1) | 89.8 (12.1) | 89.7 (13.3) | 90.1 (11.6) |
| Hip circumference (cm) | 103.2 (9.8) | 103.2 (9.9) | 101.7 (10.7) | 104.4 (9.4) |
| Waist/hip ratio | 0.87 (0.08) | 0.87 (0.08) | 0.88 (0.08) | 0.86 (0.08) |
| **Biomarkers (geometric mean (geometric SD))** |  |  |  |  |
| IL-6 (pg/ml) | 0.72 (2.36) | 0.73 (2.39) | 0.61 (1.98) | 0.79 (1.99) |
| IL-8 (pg/ml) ^c^ | 8.71 (1.93) | 8.97 (2.01) | 9.80 (2.09) | 9.30 (1.95) |
| IL-10 (pg/ml) | 0.21 (1.83) | 0.20 (1.81) | 0.21 (2.14) | 0.22 (2.15) |
| TNF-α (pg/ml) | 1.95 (1.54) | 1.92 (1.53) | 2.02 (1.68) | 2.25 (1.60) |
| IFN-γ (pg/ml) | 4.11 (2.26) | 4.10 (2.29) | 4.27 (2.09) | 4.99 (2.84) |
| Leptin (ng/ml) ^d^ | 6.73 (4.45) | 6.63 (4.42) | 5.81 (4.34) | 2.99 (5.53) |
| Adiponectin (µg/ml) | 11.86 (2.42) | 11.9 (2.46) | 11.73 (2.84) | 10.35 (2.32) |
| Leptin/adiponectin ratio | 1.75 (6.04) | 1.78 (6.04) | 2.01 (7.4) | 3.64 (6.75) |
| Abbreviations: ER Estrogen receptor; HER2 human epidermal growth factor receptor 2; IFN-γ interferon γ; IL-6 interleukin 6; IL-8 interleukin 8; IL-10 interleukin 10; PR progesterone receptor; SD standard deviation; TNF-α tumor necrosis factor α.  ^a^In parous women only. | | | | |
